# A hybrid approach for named entity recognition in Chinese electronic medical record

**DOI:** 10.1186/s12911-019-0767-2

**Published:** 2019-04-09

**Authors:** Bin Ji, Rui Liu, Shasha Li, Jie Yu, Qingbo Wu, Yusong Tan, Jiaju Wu

**Affiliations:** 1 College of Computer, National University of Defense Technology, Changsha, China; 2 Department of Oncology, the Second Xiangya Hospital of Central South University, Changsha, China; 3 Institute of Computer Application, China Academic of Engineering Physics, Mianyang, China

**Keywords:** BiLSTM-CRF, Attention, Chinese electronic medical record, Named entity recognition, Drug dictionary

## Abstract

**Background:**

With the rapid spread of electronic medical records and the arrival of medical big data era, the application of natural language processing technology in biomedicine has become a hot research topic.

**Methods:**

In this paper, firstly, BiLSTM-CRF model is applied to medical named entity recognition on Chinese electronic medical record. According to the characteristics of Chinese electronic medical records, obtain the low-dimensional word vector of each word in units of sentences. And then input the word vector to BiLSTM to realize automatic extraction of sentence features. And then CRF performs sentence-level word tagging. Secondly, attention mechanism is added between the BiLSTM and the CRF to construct Attention-BiLSTM-CRF model, which can leverage document-level information to alleviate tagging inconsistency. In addition, this paper proposes an entity auto-correct algorithm to rectify entities according to historical entity information. At last, a drug dictionary and post-processing rules are well-built to rectify entities, to further improve performance.

**Results:**

The final F1 scores of the BiLSTM-CRF and Attention-BiLSTM-CRF model on given test dataset are 90.15 and 90.82% respectively, both of which are higher than 89.26%, which is the best F1 score on the test dataset except ours.

**Conclusion:**

Our approach can be used to recognize medical named entity on Chinese electronic medical records and achieves the state-of-the-art performance on the given test dataset.

## Background

Named Entity Recognition (NER) is a basic task in Natural Language Processing (NLP). Its purpose is to recognize naming mentions from text, paving the way for tasks such as relation extraction. In a narrow sense, NER is to recognize three kinds of named entity, which are name, place, and organization [1]. In medical field, with rapid development of electronic medical records and clinical information, doctors need information-based means to obtain patient-related information from a large number of electronic medical records (EMRs) quickly and accurately, to improve work efficiency. There are two main types of EMRs, which are outpatient medical records and inpatient medical records. Outpatient medical records are usually short, containing less information, and doctors can easily obtain required information from them; Inpatient medical record includes numerous information, e.g., hospital records, progress note, order sheet, case data, etc.. Among them, progress note is the key part, which focuses on the occurrence, evolution and treatment of patient’s existing diseases, including plenty of medical entities and is the key research content of EMRs. Today, it is still a huge challenge for NER in Chinese EMRs, due to the following reasons; firstly, there is no uniform standard to name medical entities. Different hospitals and even different doctors may name the same entity differently; secondly, there may be several names for one entity, e.g. a drug can have tens of trade names; thirdly, new entities are constantly being created; last but not least, usage of Chinese is flexible. Some words cannot be judged as named entities without context, and there is no space between Chinese characters as boundary mark.

In previous NER tasks, the BiLSTM-CRF, which is the abbreviation of bi-directional Long-Short Term Memory (LSTM) joining with a conditional random field (CRF) layer, based approach exhibits the best performance [2, 3], and is the prevalent approach to NER tasks. Compared to CRF based approach, there is no need to edit complex feature templates manually in this approach; instead features can be extracted by LSTM automatically. However, although LSTM can preserve long time information through gate mechanism [4], it still leads to recognition error in long sentences [5], which is defined as tagging inconsistency in [6]. Attention mechanism can be used to solve tagging inconsistency, which has been widely used in various fields of deep learning recently, e.g., image processing, speech recognition, NLP, etc. [7, 8]. More recently, Luo [6] et al. solves the tagging inconsistency problem in chemical NER by adding attention mechanism in BiLSTM-CRF model.

Our work focus on NER in Chinese EMRs, which has been subtask of several public conferences in medical domain, e.g. China Conference on Knowledge Graph and Semantic Computing (CCKS), and China Health Information Processing Conference (CHIP). These tasks not only accelerate the research of NER on Chinese EMRs, but also provide several precious corpuses for Chinese clinical entity recognition. In this paper, firstly, we realize medical NER on Chinese EMRs with BiLSTM-CRF model. And then, we construct Attention-BiLSTM-CRF model by adding Attention mechanism into BiLSTM-CRF model to alleviate tagging inconsistency problem and promote system performance. Our Contributions are summarized as follows.

- We realize medical NER in Chinese EMRs with BiLSTM-CRF model. And by adding attention mechanism into BiLSTM-CRF, we construct Attention-BiLSTM-CRF model and apply it to NER in Chinese EMRs, which aims at alleviating tagging inconsistency problem by leveraging document-level information. As far as we know, we are the first to apply Attention-BiLSTM-CRF model to medical NER in Chinese EMRs.
- We propose an entity auto-correct algorithm, which depends on historical entity information, to automatically rectify entity if necessary. In addition by collecting medical information, we built a drug dictionary to assist drug entity recognition. The drug dictionary basically covers all medicines currently on the market, including their product names and trade names. At last, By analyzing the recognition results, we edit universal post-processing rules to rectify entity boundary partition error and extract entities that cannot recognized by neural network model.

Owning to the contributions above, our method achieves the state-of-the-art performance for medical NER task in the Chinese EMRs, which are provided by CCKS 2018.

## Related research

NER has become an important research field in information extraction, data mining and NLP [9]. NER’s development basically experienced a shift from rules to statistics, which mainly covers the following three approaches.

### Rule based approach

Hand-written rules are used to match text to extract named entities. For example, for Chinese EMRs, words, e.g., “术” (“surgery”) and “手术” (“surgery”), can be used as the end of surgery entity; words, e.g., “炎” (“inflammation”) and “癌” (“cancer”), can be used as the direct next word of anatomy entity. Rule construction often requires professional linguistic knowledge, and rule confliction need to be handled with caution. In addition, rules are hard to generalize from one domain to others.

### Feature template based approach

The statistical machine learning method treats NER as a sequence tagging task, and uses a large-scale corpus to learn tagging model [10, 11]. Models used in NER tasks include generated model (e.g. HMM), discriminant model (e.g. CRF), etc. The most prevalent method is the “feature template + CRF” scheme: the feature template is usually some manually defined binary feature functions, which try to mine the internal characteristics of named entity and context. Different feature templates can be combined to form a new feature template. The advantage of CRF is that it can use the information already generated in the process of tagging a sequence, and use Viterbi decoding to get the optimal sequence.

### Neural network based approach

Recently, with the development of hardware capabilities and the emergence of word embedding, neural network model can effectively handle many NLP tasks. This type of model maps words from discrete one-hot representations to low-dimensional and dense word embeddings, then inputs sentence’s embedding sequence into recurrent neural network (RNN) to extracts features automatically, and predicts each words tag with Softmax function. This method makes model training an end-to-end process, rather than traditional pipeline process, which is a data-driven method. However, there are many variants of neural networks, which depend on parameter settings severely, and neural network model is poor interpretability.

More recently, researchers proposed LSTM-CRF model for sequence tagging, which is a combination of feature-template based approach and neural network based approach. The LSTM-CRF approach exhibits the state-of-the-art results in many NLP tasks. Collobert [12] et al. firstly put forward the concept of joining CRF model with LSTM model. Huang [13] and Lample [14] took LSTM-CRF to make sentence-level tag predictions, which made the tagging process no longer independent of each token. Ma [15] et al. introduced LSTM to English NER task. Dong [16] et al. firstly applied LSTM-CRF to Chinese NER task.

## Dataset, entity definition and corpus

The training data and test data used in this paper come from CCKS2018, and are jointly provided by Tsinghua University Knowledge Engineering Laboratory and Yiducloud (Beijing) Technology Co., Ltd. Training data consists of 600 copies Chinese EMRs, each of which consists of two parts: one raw Chinese EMR and corresponding tagged entities. Tagged entity is tagged manually according to specific application requirements, which includes entity name, entity start position, entity end position, and entity category. The test data consists of 400 raw Chinese EMRs.

In this paper, the goal of NER in Chinese EMRs is to recognize five categories of entity, which are anatomy, surgery, drug, independent symptom and symptom description. And the recognized entities should be organized into items as the example shows below.

- “胃 (stomach) 12 13 解剖部位 (anatomy)”.
- “胃 (stomach)” in the item is entity name, “12” represents the entity start position in EMR, while “13” represents the end position, and “解剖部位 (anatomy)” represents the entity category. These four parts of the item are separated by tab. Definition rules of the five categories of entity are shown below.
  1. *Anatomy*: A structural function unit that is composed of a variety of tissues, e.g. “腹部” (abdomen).
  2. *Symptom description*: refers to patient’s experience and feeling of abnormal physiological function when the patient is ill. At the same time, it needs to be output separately from anatomy, e.g. “腹部不适” (“abdomen discomfort”), “腹部” (“abdomen”) and “不适” (“discomfort”) need to be output as anatomy and symptom description respectively.
  3. *Independent symptom*: refers to the self-experience and feeling of patient’s physiological function when the patient is ill, e.g. “眩晕” (“dizziness”).
  4. Drug: chemicals used to treat, prevent diseases, or promote health.
  5. Surgery: refers to treatment of patient’s body with medical devices, e.g. resection, suturing, etc.

**Fig. 1:**
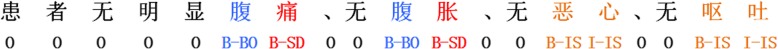
BIO tagging schema

According corresponding tagged entities, Chinese EMRs are encoded with BIO (Begin, Inside, Outside) tagging schema to construct training corpus. Among them, B-BO and I-BO represent the beginning and inside word of anatomy respectively. Similar to anatomy, B-SU and I-SU represent surgery, B-DR and I-DR represent drug, B-SD, I-SD represent symptom description, and B-IS and I-IS represent independent symptom; O means that the word does not belong to any entity. Figure 1 gives an example of a BIO tagging schema.

Here, we’d like to address problems of the dataset provided by CCKS. Entities in Chinese EMRs are very complex and in many cases it is difficult to find a universal standard to define. Therefore, entities tagged manually inevitably have tagging errors made by human. As far as we know, the training dataset contains a total of 15,080 entities, most of which are uncontroversial, and the remaining few is uncertain, which are regarded as noise entities.

Similar to the tagging inconsistency problem described in [6], the tagging inconsistency of medical NER on Chinese EMRs is shown below. Take “**肝S2, 3**虑转移瘤, 较前缩小。2016年03月16日在我院行扩大左半肝切除术, 术后病理:1(S2, 3肿物)病灶减小…”, which is an segment of Chinese EMR, as an example. The mentions in bold type can be recognized by BiLSTM-CRF model. For convenience, doctors may abbreviate “肝S2, 3” for “S2, 3” or some other forms, e.g. “S2”, “S3”, etc. Reasonably, these mentions should be tagged with the same tags. However, the mention “S2, 3” with an underline cannot be recognized by the model. There are many similar situations in Chinese EMRs, so it is an important factor affecting model performance.

## Method

In this section, we first introduce the architecture of neural network based approach to medical NER in Chinese EMRs. Then BiLSTM-CRF model and Attention-BiLSTM-CRF model are introduced respectively. And then we introduce the entity auto-correct algorithm. At last, drug dictionary and post-processing rules are introduced.

### Architecture of neural network based approach

**Fig. 2:**
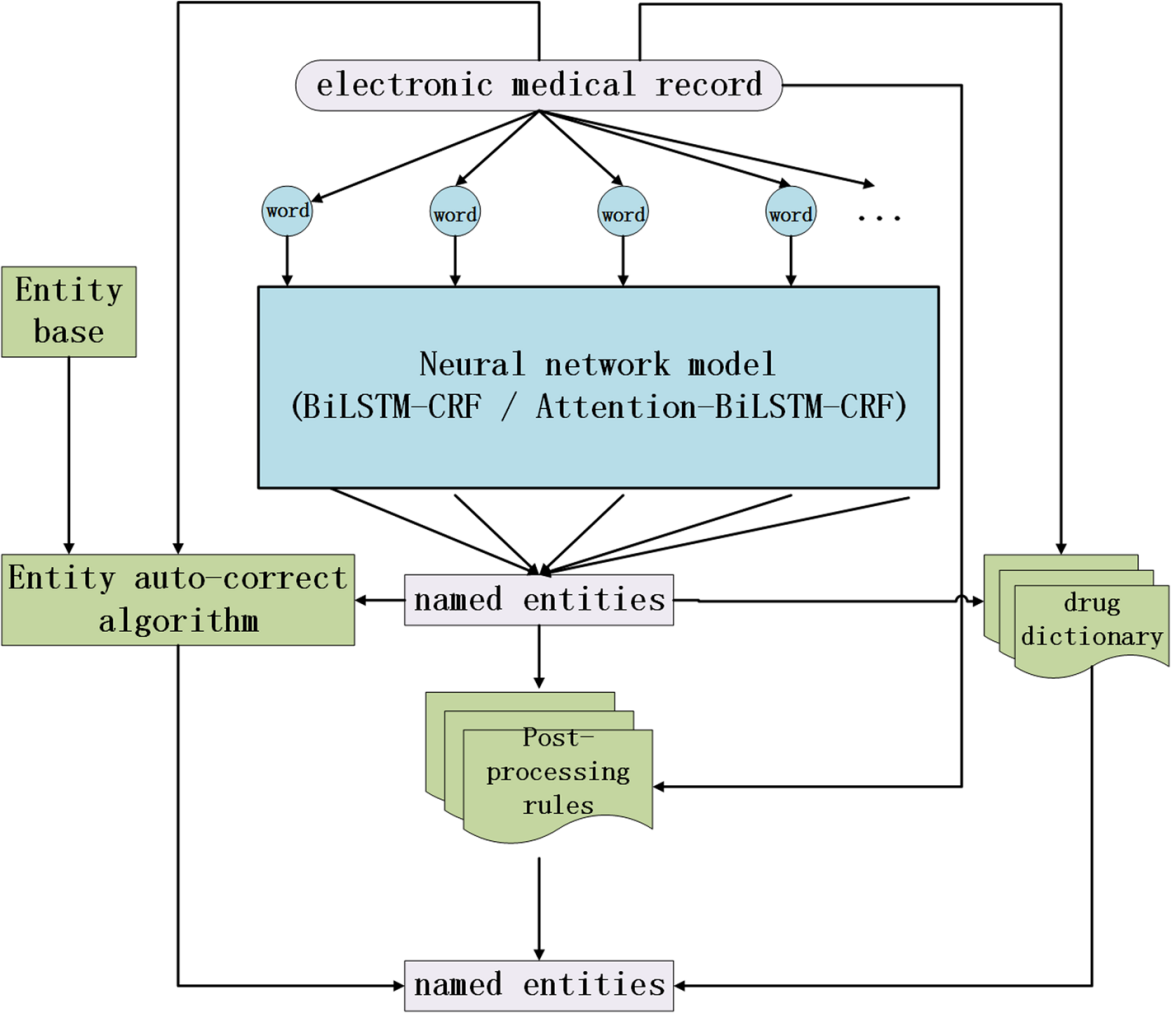
Architecture of neural network based approach to medical NER on Chinese EMRs

The architecture diagram of neural network based approach to medical NER on Chinese EMRs is shown in Fig. 2. In this approach, neural network, which is BiLSTM-CRF or Attention-BiLSTM-CRF, are taken to recognize the five categories of entity from Chinese EMRs. In addition, we added three auxiliary measures to improve entity recognition accuracy. The auxiliary measures are entity auto-correct algorithm, drug dictionary and post-processing rules, which will be introduced in detail in the following parts respectively.

### BiLSTM-CRF model

**Fig. 3:**
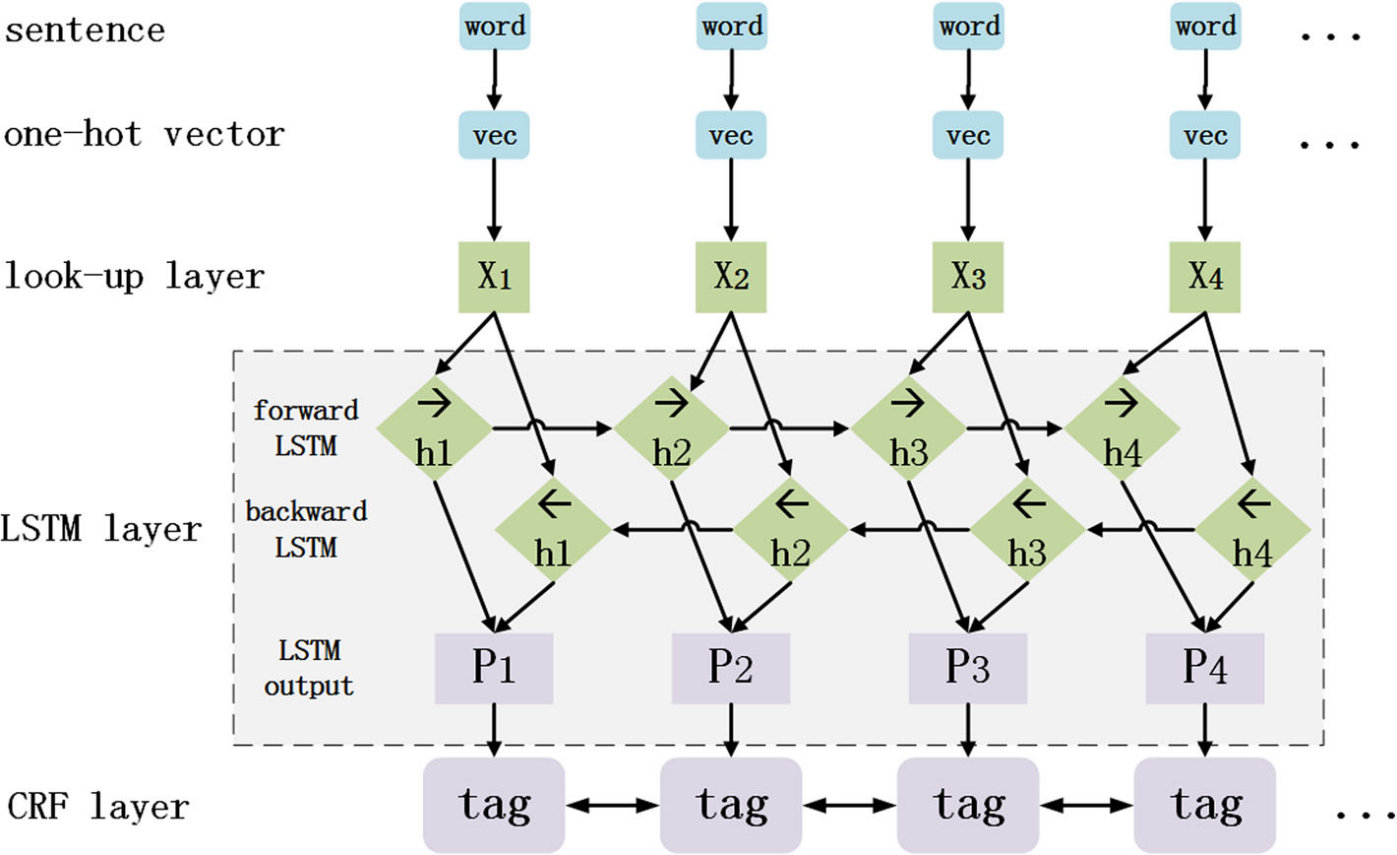
Architecture of BiLSTM-CRF model

The architecture of BiLSTM-CRF model is shown in Fig. 3, which is similar to the classical ones described in paper [15, 17].

The BiLSTM-CRF model records a sentence containing n words as x = (x_1_,x_2_,…,x_n_) in units of sentences (sentence-level). Where x_i_ represents the id of the i^th^ word of the sentence in word dictionary, and thus the one-hot vector of each word can be obtained, and the vector dimension is dictionary size.

The first layer of the model is the look-up layer, which uses pre-trained or randomly initialized embedding matrix to map each word x_i_ in the sentence from a one-hot vector to a low-dimensional dense word vector (word embedding) x_*i*_ ∈ *R*^*d*^, d is the dimension of word embedding. In this paper, the pre-trained embedding matrix is trained with corpus from China Daily, which contains about 2.3 million words. Dropout is set to alleviate overfitting.

The second layer of the model is the bidirectional LSTM layer, which automatically extracts sentence features. The word embedding sequence (x_1_, x_2_,  … , x_*n*_) of a sentence is taken as input of each time step of the bidirectional LSTM, and the implicit state output sequence$\left(\overrightarrow{{\mathrm{h}}_1},\overrightarrow{{\mathrm{h}}_2}\dots ..,\overrightarrow{{\mathrm{h}}_{\mathrm{n}}}\right)$ $\left(\overrightarrow{{\mathrm{h}}_1},\overrightarrow{{\mathrm{h}}_2}\dots ..,\overrightarrow{{\mathrm{h}}_{\mathrm{n}}}\right)$ $\left(\overrightarrow{h_1},\overrightarrow{h_2}\dots ..,\overrightarrow{h_n}\right)$ of forward LSTM and the output sequence $\left(\overleftarrow{h_1},\overleftarrow{h_2},\dots, \overleftarrow{h_n}\right)$ of reverse LSTM are concatenated to get ${h}_t=\left[\overrightarrow{h_t};\overleftarrow{h_t}\right]\in {R}^m$, and get the complete hidden state sequence of the sentence, which can be represented by (h_1_, h_2_, ⋯, h_*n*_) ∈ *R*^*n* × *m*^.

Then a linear layer is set to map the hidden state vector from m-dimension to k-dimension (k is the number of tags defining in the tagging set), and then the automatically extracted sentence features are obtained, which are recorded as the matrix P = (p_1_, p_2_,  … , p_*n*_) ∈ *R*^*n* × *k*^. Each element p_*ij*_ of p_*i*_ ∈ *R*^*k*^ can be regarded as a score that tag the word x_*i*_ with the j^*th*^ tag. Next, a CRF layer is set to tag words.

The third layer of the model is CRF layer, which performs sequence-level word tagging. The parameter of CRF layer is a transition matrix A with a dimension of (k + 2) × (k + 2), and A_*ij*_ represents the transition score from the i^*th*^ tag to the j^*th*^ tag, so tags that have been previously tagged can be utilized when tagging a new word. A tag sequence can be represented by y = (y_1_, y_2_,  … , y_*n*_), while n equals sentence length, the formula used to measure that tag of sentence *X* equal to tag sequence y is shown in formula (1).

1 $$ \mathrm{score}\left(\mathrm{X},\mathrm{y}\right)=\sum i=1n+1{A}_{y_{i-1,}{y}_i}+\sum i=1n{P}_{i, yi} $$

It can be seen that the score(X, y) equals the sum of scores of all words in sentence and each score consist of two parts, the first part is from the transition matrix A, and the second part is from the matrix P described above. A Softmax function is then used to normalize probability, which is shown in formula (2).

2 $$ \mathrm{P}\left(\mathrm{y}|\mathrm{X}\right)=\frac{\exp \left(\mathrm{score}\left(\mathrm{X},\mathrm{y}\right)\right)}{\sum_{{\mathrm{y}}^{\prime }}\ \exp \left(\mathrm{score}\left(\mathrm{X},{\mathrm{y}}^{\prime}\right)\right)} $$

While training, for training sample (X, y^x^) formula (3) can be taken as the log probability formula to maximize the log probability of tag sequence.

3 $$ \mathrm{logP}\left({\mathrm{y}}^{\mathrm{x}}|\mathrm{X}\right)=\mathrm{score}\left(\mathrm{X},{\mathrm{y}}^{\mathrm{x}}\right)-\log \left({\sum}_{{\mathrm{y}}^{\prime }}\ \exp \left(\mathrm{score}\left(\mathrm{X},{\mathrm{y}}^{\prime}\right)\right)\right) $$

During the encoding process, Viterbi algorithm is used to calculate the optimal tag path with dynamic planning, as formula (4) shows.

4 $$ {\mathrm{y}}^{\ast }=\arg \underset{\mathrm{y}^{\prime }}{\max }\ \mathrm{score}\left(\mathrm{X},{\mathrm{y}}^{\prime}\right) $$

### Attention-BiLSTM-CRF model

**Fig. 4:**
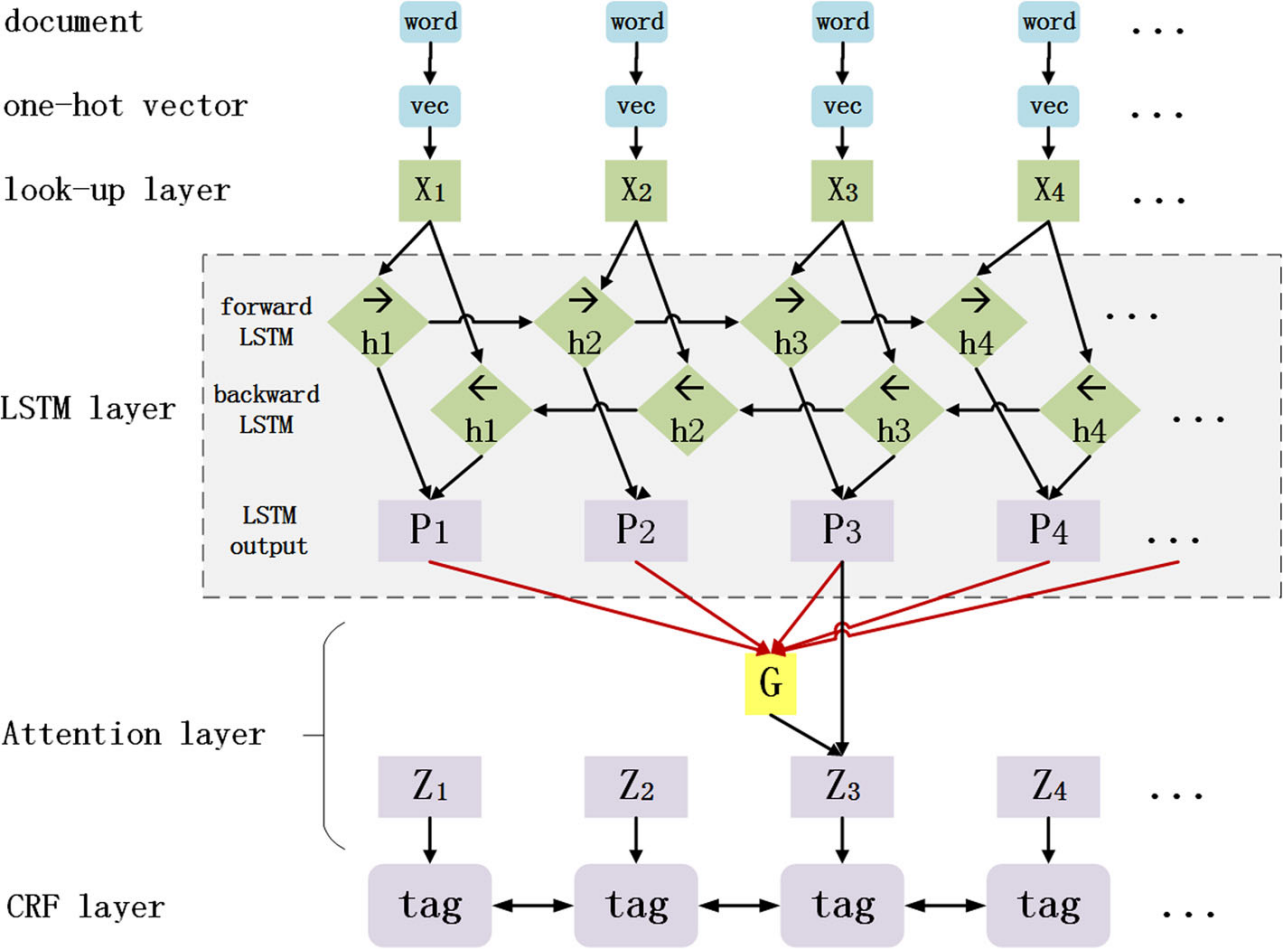
Architecture of Attention-BiLSTM-CRF model

As described above, BiLSTM-CRF is a sentence-level NER method. Although LSTM can preserve long time information through gate mechanism, it still leads to tagging inconsistency in long sentences for the later words in sentence are more dominant than former words. Inspired by Luo [6], in this paper, we construct Attention-BiLSTM-CRF model to alleviate tagging inconsistency described above. The architecture of Attention-BiLSTM-CRF model is shown in Fig. 4.

The difference between our Attention-BiLSTM-CRF model and others is that the attention mechanism in our model is used to capture related word tagging information of document-level to keep word tagging consistency.

A Chinese EMR as input document can be described as D = (X_1_, X_2_,  … , X_*m*_), where X_*i*_ (i ∈ 1, 2 … m) represent m sentences that consist of the EMR. And each sentence can be represented as X = (x_1_, x_2_,  … , x_*n*_), where n is the length of sentence X. Besides, N is defined as the number of words in the EMR. The way to obtain word embedding is just the same as the way described in BiLSTM-CRF model. And then, an attention layer is added between the BiLSTM layer and CRF layer to construct Attention-BiLSTM-CRF model. In the attention layer, matrix A is defined as attention matrix to record the similarity between the current word and all words in the EMR. The element of matrix A, which can be described as *a*_*i*, *j*_, can be calculated by formula (5).

5 $$ {a}_{i,j}=\frac{\exp \left(\mathrm{score}\left({x}_i,{x}_j\right)\right)}{\sum k=1N\exp \left(\mathrm{score}\left({x}_i,{x}_k\right)\right)} $$

Here, the similarity between x_*i*_ and x_*j*_ is calculated by Euclildean distance, as formula (6) shows.

6 $$ \mathrm{score}\left({x}_{i,}{x}_j\right)=\mathrm{W}{\left({x}_i-{x}_j\right)}^T\left({x}_i-{x}_j\right) $$

W is a weight matrix, and is set as weight parameter. According to formula (6), the attention matrix A can be calculated. Then a document-level global vector *G* can be computed by formula (7), where A is attention matrix and H is the output of BiLSTM layer, i.e. H = (h_1_, h_2_,  … , h_*n*_) .

7 $$ \mathrm{G}=\mathrm{AH} $$

The document-level vector *G* and the BiLSTM output H are concatenated as a vector Z, which is the input of a tanh function. And output of the tanh function is the input of the CRF layer.

At last, the CRF layer is set to perform word tagging. For an input Chinese EMR, the score that the EMR is tagged by tag path y is calculated by formula (8), where P is the output of tanh function described above, A is the transition matrix of CRF layer and m is the number of sentences in EMR.

8 $$ \mathrm{s}\left(\mathrm{EMR},\mathrm{y}\right)=\sum j=1m\sum i=1n\left({A}_{y_{i-1},{y}_i}+{P}_{i,{y}_i}\right) $$

The following process is just the same as the BiLSTM-CRF model does.

### Entity auto-correct algorithm

Besides tagging inconsistency, entity boundary partition error is another dominating defect in both BiLSTM-CRF and Attention-BiLSTM-CRF based approach. Some examples of entity boundary partition error are shown in Table 1.

**Table 1:** Examples of entity boundary partition error

Recognized entity	Correct entity	Category
左附件 (left attachment)	左附件区 (left attachment area)	Anatomy
卵巢切除 (Ovariectomy)	卵巢切除术 (Ovariectomy surgery)	surgery
贝伐 (Beval)	贝伐珠单抗 (Bevacizumab)	Drug
睡眠不佳 (Poor sleep)	饮食睡眠不佳 (poor diet and sleep)	Individual symptom
疼痛 (pain)	疼痛不适 (pain discomfort)	Symptom description

In order to alleviate entity boundary partition error, we proposed an entity auto-correct algorithm, which can rectify entity boundary according to entity history information. The brief description of the algorithm is shown in Table 2.

**Table 2:**
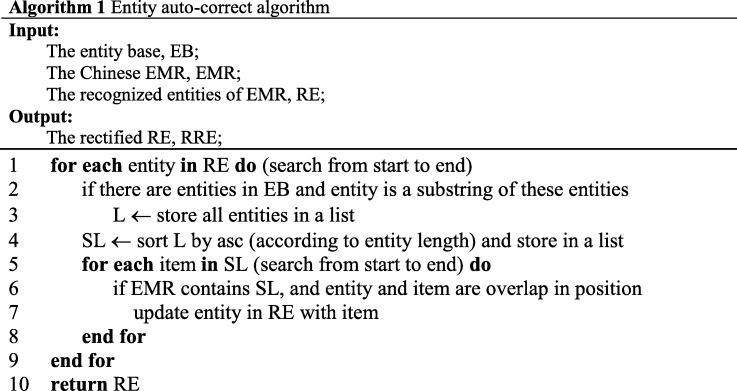
Entity auto-correct algorithm

The entity base is built with entities from training dataset. The item in entity base is in form of “entity category occurrence”, “entity” in is the entity name, and “category” is entity category, e.g. anatomy, surgery, etc., and “occurrence” is the times that the entity appears in training dataset. In short, entity base is a statistical result of all entities existing in training dataset.

In this algorithm, we assume that all entity recognition results in the training dataset are correct and there is no noise data. And we assume that for the same entity, the tags in the training dataset and the test dataset is identical.

### Drug dictionary

Drug name can be mainly divided into drug product name and drug trade name. Drug with the same product name but made by different manufacturers may have different trade names. In addition, anti-tumor drugs generally appear in the form of English abbreviations in Chinese EMRs, e.g. “5-FU”, “5FU” and the like. Under condition of training dataset is small and drug name representations are diverse, a well-built drug dictionary can both rectify error drug entity and extract drug entity that cannot be recognized by neural network model, which can effectively improve drug entity recognition performance.

National Market Supervisory Authority gives product names of 17,972 domestic drugs and 1361 imported drugs, and 7720 of which have trade names [18]. In addition, trade names of other 3233 drugs and English abbreviations of 577 anti-tumor drugs were obtained by processing numerous medical literature and textbooks. Based on the obtained data, a drug dictionary is well-built and used as an auxiliary measure for drug entity recognition.

The drug entity extraction process with drug dictionary is not affected by the drug entity recognition process performed by neural network model, which can be regarded as a two-factor authentication for drug entity. For drug entity contained in drug dictionary, the drug dictionary can rectify drug entity boundary partition errors and extract drug entity that cannot be recognized by neural network model. In particular, it should be noted that when taking the drug dictionary into practice, drug names inclusion relationships should be paid more attention, e.g. the two drugs, CPT-11″ and “CPT”. When “CPT-11” appears in EMR, “CPT-11” should be recognized as a drug entity rather than “CPT”.

### Post-processing rule

In order to further improve entity recognition performance, aiming at dominating defects that exist in neural network based approach, we edit post-processing rules. The practical effect of post-processing rules may partially reduplicate with the entity auto-correct algorithm mentioned above. Post-processing rules can be divided into the following two types.

#### Rules to rectify entity boundary partition error

Some example rules are shown below.

6. Remove the word “旁” (“side”) from recognized anatomy entities, which are in form of “xxxx旁”;
7. If the phrase before recognized anatomy entity represents orientation, e.g., “右” (“right”), “右上” (“upper right”), “左” (“left”) and “左上” (“upper left”), etc., then combine the orientation phrase with anatomy entity to form a new anatomy entity.
8. If the phrase after recognized anatomy entity represents orientation, e.g., “周” (“around”), “上” (“up ”), “下” (“down”) and “外” (“outside”), etc., then combine anatomy entity with the orientation phrase to form a new anatomy entity.
9. If the phrase after recognized surgery entity is “术” (“surgery”) or “手术” (“surgery”), then combine surgery entity with the phrase to form a new surgery entity.

#### Rules to extract the entities that cannot be recognized by neural network model

In order to achieve this goal, we create an entity base, which contains all the entities in training dataset, and entities extracted from medical literature, web medical resource, etc. Entity base mainly contains two kinds of entity, which are anatomy and surgery. The function of entity base is similar to the second function of drug dictionary, which is used to extract the entities that cannot be recognized by neural network model.

## Results and discussion

Firstly, the training corpus is used to train both BiLSTM-CRF and Attention-BiLSTM-CRF model. During training process, batch_size takes 1 and “Adam” is used as optimizer to train 45 epochs for the two models respectively. The number 45 is an empirical optimal value, which is obtained through plenty of experiments. The test results of the two neural network models on the given test dataset are shown in Table 3, which are provided by the CCKS 2018 evaluation platform [19], and the definition of strict index can be found on this evaluation platform, too.

**Table 3:** Test results of the two neural network models on given dataset

Entity name	BiLSTM-CRF			Attention-BiLSTM-CRF		
	Strict index (%)			Strict index (%)		
	P	R	F1	P	R	F1
Anatomy	85.57	85.61	85.59	86.29	86.24	86.27
Surgery	85.81	85.58	85.69	86.19	84.90	85.54
Drug	94.92	78.11	85.70	89.73	85.98	87.81
Independent symptom	92.45	89.52	90.96	91.93	90.20	91.05
Symptom description	91.81	87,91	89.82	91.58	87.69	89.59
Total	87.66	85.72	86.68	87.75	86.77	87.26

As shown in Table 3, the F1 score of Attention-BiLSTM-CRF model is about 0.58% higher than the F1 score of BiLSTM-CRF model. And the recognition result shows that 68 entities are rid of tagging inconsistency because of the attention layer added in Attention-BiLSTM-CRF model.

And then we take the drug dictionary as an auxiliary measure in both models. Test results of the two models with drug dictionary (abbreviated as dictionary) are shown in Table 4.

**Table 4:** Test results of the two models with drug dictionary

Approach	Strict index (%)		
	P	R	F1
BiLSTM-CRF	87.66	85.72	86.68
BiLSTM-CRF + dictionary	88.61	86.83	87.71
Attention-BiLSTM-CRF	87.75	86.77	87.26
Attention-BiLSTM-CRF + dictionary	88.79	87.80	88.29

From Table 4 we can get that with drug dictionary, F1 scores of both models are about 1% higher than before, which means that about 100 drug entities are rectified or extracted with the help of drug dictionary.

Next step, we take the post-processing rules as an auxiliary measure in both models. Test results of the two models with post-processing rules are shown in Table 5.

**Table 5:** Test results of the two models with post-processing rules

Approach	Strict index (%)		
	P	R	F1
BiLSTM-CRF	87.66	85.72	86.68
BiLSTM-CRF + rule	89.43	87.47	88.44
Attention-BiLSTM-CRF	87.75	86.77	87.26
Attention-BiLSTM-CRF + rule	89.61	88.58	89.09

From Table 5 we can get that with the post-processing rules, F1 scores of both models are about 1.7% higher than before, which means about 170 entities are rectified or extracted.

Next step, we take the entity auto-correct algorithm as an auxiliary measure in both models. Test results of the two models with entity auto-correct algorithm (abbreviated as algorithm) are shown in Table 6.

**Table 6:** Test results of the two models with entity auto-correct algorithm

Approach	Strict index (%)		
	P	R	F1
BiLSTM-CRF	87.66	85.72	86.68
BiLSTM-CRF + algorithm	88.73	86.78	87.74
Attention-BiLSTM-CRF	87.75	86.77	87.26
Attention-BiLSTM-CRF + algorithm	88.72	87.71	88.21

As shown in Table 6, with the auto-correct algorithm, F1 scores of both models are about 1% higher than before, which is almost the same as the performance improvement created by drug dictionary but is lower than the performance improvement created by post-processing rules.

At last, we add all the three available auxiliary measures to both models. Test results of the two models with all auxiliary measures are shown in Table 7.

**Table 7:** Test results of the two models with all auxiliary measures

Approach	Strict index (%)		
	P	R	F1
BiLSTM-CRF	87.66	85.72	86.68
BiLSTM-CRF + all	91.12	89.21	90.15
Attention-BiLSTM-CRF	87.75	86.77	87.26
Attention-BiLSTM-CRF + all	91.26	90.38	90.82

Table 7 gives the final results of the two neural network based approach to medical NER in Chinese EMRs, and the F1 scores of the two neural network based approach are 90.15 and 90.82% respectively, which are about 3.5% higher than the original results shown in Table 3. But 3.5% is lower than the sum of 1, 1.7, 1%, which are created by the three auxiliary measures respectively. The reason is that the three auxiliary measures act on some common entities. The F1 score of 90.82% is about 1.6% higher than the F1 score of 89.26%, which was created by the best team that participated track I of CCKS2018 and the F1 score of 90.82% is the state-of-the-art value to our knowledge.

## Conclusion

In this paper, we firstly apply the BiLSTM-CRF model to medical NER on Chinese EMRs. And then attention mechanism is added to BiLSTM-CRF model to construct Attention-BiLSTM-CRF model, which aims at alleviating tagging inconsistency problem. For the defects of the entity boundary partition error, entity recognition incomplete and other dominating ones, drug dictionary, post-processing rules and entity auto-correct algorithm are taken as auxiliary measures to alleviate these defects. Under the strict index, the final F1 score of the BiLSTM-CRF and Attention-BiLSTM-CRF model on given test data are 90.15 and 90.82% respectively. Both of the two F1 scores are higher than the best F1 score of CCKS track I, and our approach achieves the state-of-the-art performance to our knowledge.

## Abbreviations

CCKS: China Conference on Knowledge Graph and Semantic Computing
CHIP: China Health Information Processing Conference
CRF: Conditional random field
EMR: Electronic medical record
LSTM: Long-Short Term Memory
NER: Named entity recognition
NLP: Natural Language Processing
